# Prognostic factors for pediatric patients with severe intestinal motility disorders: a single institution’s experience

**DOI:** 10.1007/s00595-024-02910-1

**Published:** 2024-08-07

**Authors:** Keisuke Yano, Mitsuru Muto, Koshiro Sugita, Masakazu Murakami, Shun Onishi, Toshio Harumatsu, Yumiko Iwamoto, Masato Ogata, Lynne Takada, Nanako Nishida, Chihiro Kedoin, Ayaka Nagano, Mayu Matsui, Koji Yamada, Waka Yamada, Makoto Matsukubo, Takafumi Kawano, Tatsuru Kaji, Satoshi Ieiri

**Affiliations:** 1 Department of Pediatric Surgery, Research Field in Medical and Health Sciences, Medical and Dental Area, Research and Education Assembly, Kagoshima University, Kagoshima, Japan; 2 Department of Pediatric Surgery, Kurume University School of Medicine, Kurume, Japan

**Keywords:** Intestinal motility disorder, Intestinal-associated liver disease, Cholestasis, Catheter-related blood stream infection, Prognostic factor

## Abstract

**Purpose:**

To identify the prognostic factors for pediatric severe intestinal motility disorder (IMD).

**Methods:**

We reviewed the medical records of patients with severe IMD, who required total parenteral nutrition (TPN) for ≥ 60 days at our institution between April, 1984 and March, 2023, examining their characteristics to identify prognostic factors.

**Results:**

The types of IMD in the 14 patients enrolled in this study were as follows: isolated hypoganglionosis (IHG, *n* = 6), extensive aganglionosis (EAG: *n* = 6), and chronic idiopathic intestinal pseudo-obstruction (CIIP, *n* = 2). There was no significant difference in mortality among the three types of severe IMD. Weaning-off TPN and the use of the colon were not significant prognostic factors, but cholestasis was a significant prognostic factor (*p* = 0.005). There was a high mortality rate (50%), with the major causes of death being intestinal failure-associated liver disease (IFALD) following hepatic failure, and catheter-related blood stream infection (CRBSI). One IHG patient underwent small bowel transplantation but died of acute rejection.

**Conclusion:**

Severe IMD is still associated with a high mortality rate and cholestasis predicts the prognosis. Thus, preventing or improving IFALD and CRBSI caused by long-term TPN is important for reducing the mortality rate.

## Introduction

Intestinal failure (IF) in infants and children is caused mainly by short bowel syndrome (SBS) and severe intestinal motility disorder (IMD) [1]. Since these disorders require multidisciplinary management because of the absence of normal gastrointestinal function, it is important to understand the predictive prognostic factors that are encountered in the daily care of these patients. Previous studies have evaluated the prognostic predictors for SBS [2–4], but there is little information on those for severe IMD [5–8].

Patients with severe IMDs such as extensive aganglionosis (EAG), isolated hypoganglionosis (IHG), chronic idiopathic intestinal pseudo-obstruction (CIIP), and megacystis–microcolon intestinal hypoperistalsis syndrome (MMIHS), require long-term total parenteral nutrition (TPN) management for electrolytes and nutritional contents [9]. Despite the small number of children with severe IMDs in Japan limiting the number of cases experienced at each institution, based on our experience, weaning from TPN for severe IMD was associated with more difficulties than weaning from TPN for SBS. Our severe IMD patients on long-term TPN suffered eventually from catheter-related blood stream infection (CRBSI) and intestinal failure-associated liver disease (IFALD). Moreover, the previous reports from Japan based on the national surveys have provided little information on factors associated with the survival of severe IMD patients [10–12]. Because experiences of treating severe IMD have not been shared among pediatric surgeons, a high level of evidence is lacking; yet, it is important to evaluate the actual situation objectively and to accumulate information. We conducted this to investigate the prognostic factors for severe IMD through our experience.

## Patients and methods

### Study design and data collection

We conducted a retrospective chart review of four decades from January, 1984 to March, 2023. Patients with severe IMD who required TPN for > 60 days were enrolled in this study. The following patient characteristics were evaluated: gestational age at birth, birth weight, and sex; and the association of the following factors with survival: age at enterostomy, location of enterostomy, presence or absence of weaning from TPN, surgical procedure for use of the distal intestine beyond the stoma, and history of cholestasis (direct bilirubin > 2.0 mg/dL).

### Statistical analyses

Continuous variables are described as the median and range with maximum and minimum values. Nominal variables are described as numbers and percentages. The survival time was analyzed using a log-rank test and Kaplan‒Meier curves. The survival period was defined as the time from birth to the last date of confirmed survival or mortality. P values of < 0.05 were considered to indicate significance. All statistical analyses were performed using EZR (Saitama Medical Center, Jichi Medical University, Saitama, Japan), a modified version of R commander (The R Foundation for Statistical Computing, Vienna, Austria) designed to add statistical functions that are used frequently in biostatistics [13].

### Ethical approval

This study was performed in accordance with the Ethical Guidelines for Medical and Health Research Involving Human Subjects by the Ministry of Health, Labor, and Welfare of Japan in 2014 and in compliance with the 1964 Declaration of Helsinki (revised in 2013). Patient data were collected with the blinding of personal information and registered using consecutive patient numbers. This study was approved by the local ethics committee of our institution (registration number: 210329). Informed consent for publication was obtained from the patients’ parents.

## Results

### Patient characteristics and surgical interventions

**Fig. 1:**
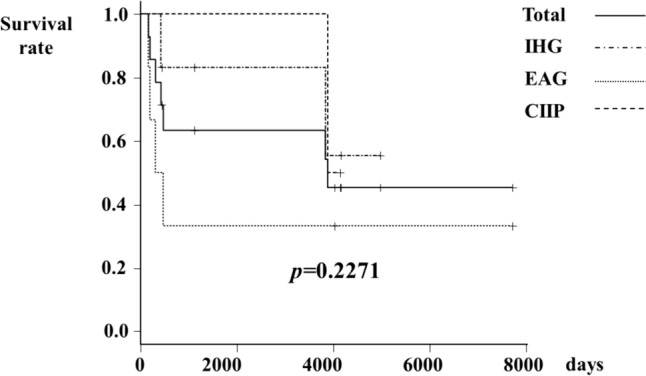
Kaplan‒Meier curve for patients with severe intestinal motility disorders

A total of 14 patients with severe IMD were included in this study. The underlying diseases were EAG (*n* = 6; 42.9%), IHG (*n* = 6; 42.9%), and CIIP (*n* = 2; 14.3%) (Table 1). The diagnosis of these three diseases was confirmed histopathologically. The diagnostic criteria for IHG and CIIP were based on the guidelines developed in Japan [12]. Table 1 summarizes the patients’ characteristics and their clinical course. The patients with EAG and IHG were mature infants born at term gestation who developed functional ileus in the neonatal period. On the other hand, the patients with CIIP developed the disease at school age. The initial surgical intervention was enterostomy in all patients. The location of enterostomy from the ligament of Treitz depended on each disease. One patient with EAG (16.7%) and one patient with IHG (16.7%) were weaned off TPN. Colon use was attempted as a surgical technique aimed at effectively utilizing the distal intestine beyond the stoma in four patients. Only one patient with IHG was treated by pull-through, while one patient with IHG and one patient with CIIP were treated with colon patch and ileostomy, one patient with IHG (14.3%) underwent treatment with the Bishop–Koop method, and one patient with IHG underwent endo-rectal pull-through using a colon patch. The overall mortality rate was 50.0% and the mortality rate for each disease was 66.3% for EAG, 33.3% for IHG, and 50.0% for CIIP. (Fig. 1).

**Table 1:** Clinical characteristics and treatment outcomes of patients with the three types of severe intestinal motility disorders

	EAG (*n* = 6)	IHG (*n* = 6)	CIIP (*n* = 2)
Gestational age at birth (weeks)	38 [37–40]	38 [36–40]	37 [36–38]
Birth weight (g)	3032 [2608–3800]	3026 [2640–3410]	1814 [1560–2068]
Sex (male:female)	3:3	4:2	0:2
Age at initial surgery of enterostomy (days)	3 [0–26]	3 [1–17]	3855.5 [3506–4205]
Location of enterostomy from ligament of Treitz (cm)	15 [7–50]	80 [5–100]	40 [10–70]
Surgical techniques for utilizing the distal intestine beyond the stoma	–	Pull-through (*n* = 1) Colon patch (*n* = 1) Bishop-Koop (*n* = 1)	Colon patch (*n* = 1)
Use of colon for a surgical technique (n, %)	0 (0.0%)	3 (50.0%)	1 (50.0%)
Weaning TPN	1 (16.7%)	1 (16.7%)	0 (0.0%)
Mortality (*n*, %)	4 (66.7%)	2 (33.3%)	1 (50.0%)

*IMD* intestinal motility disorder, *EAG* extensive aganglionosis, *IHG* isolated hypoganglionosis, *CIIP* chronic idiopathic intestinal pseudo-obstruction, *TPN* total parenteral nutrition

### Details of nonsurvivors

Table 2 summarizes the details of the nonsurvivors. The major cause of death was IFALD following the hepatic failure suffered by all nonsurvivors and the survival time was approximately 1 year. The cause of death of patients without cholestasis was sepsis caused by CRBSI and acute rejection after small bowel transplantation.

**Table 2:** Details of nonsurvivors

Patient	Sex	Type of diseases	Location of enterostomy from ligament of Treitz	Usage of colon	Cholestasis	Survival days	Cause of death
1	Female	CIIP	10	−	−	3886	Sepsis: CRBSI
2	Male	EAG	7	−	+	190	Hepatic failure caused by IFALD
3	Male	EAG	15	−	+	153	Hepatic failure caused by IFALD
4	Female	IHG	95	+	−	3827	Acute rejection after small bowel transplantation
5	Male	IHG	5	−	+	420	Hepatic failure caused by IFALD
6	Female	EAG	14	−	+	462	Hepatic failure caused by IFALD
7	Male	EAG	15	−	+	299	Hepatic failure caused by IFALD
			15 [5–95]			420 [153–3886]	

*CIIP* chronic idiopathic intestinal pseudo-obstruction, *CRBSI* catheter related blood stream infection, *EAG* extensive aganglionosis, *IHG* isolated hypoganglionosis, *IFALD* intestinal failure-associated liver disease

### Analysis of prognostic factors

**Fig. 2:**
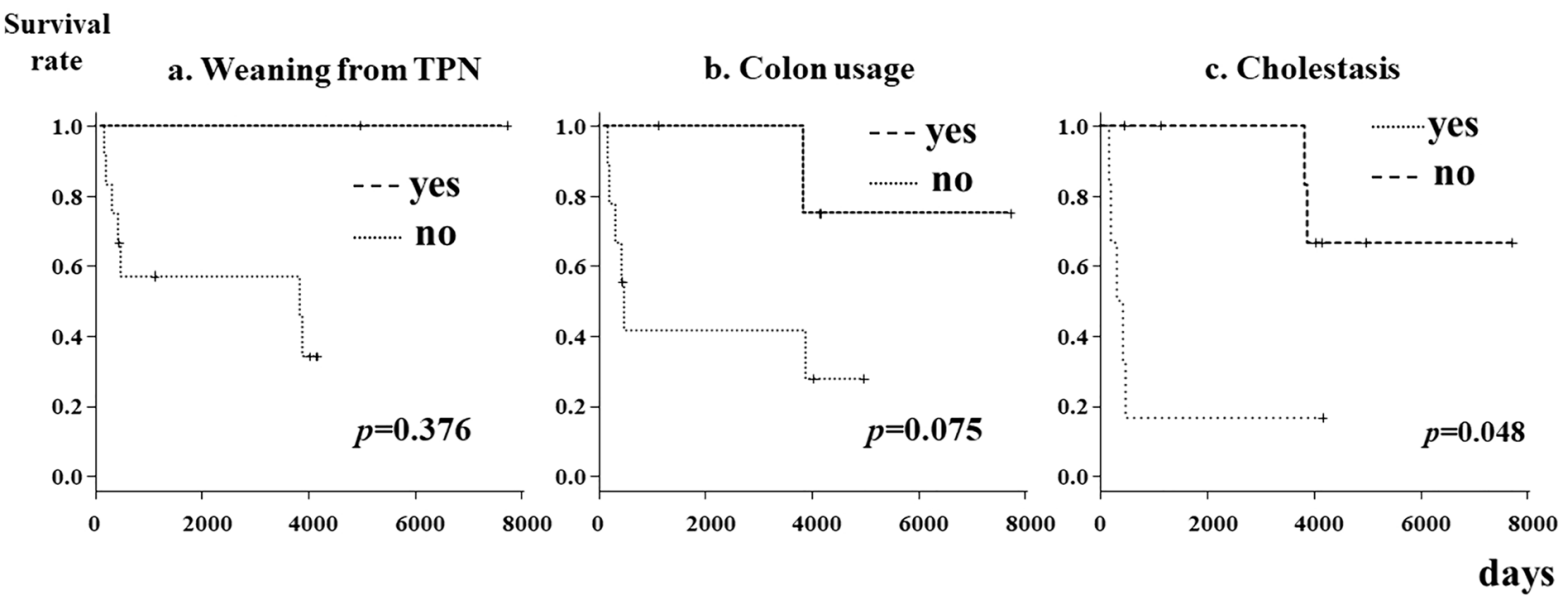
Kaplan‒Meier curves for prognostic factors in patients with severe intestinal motility disorders

A log-rank test was used to perform a time-to-event analysis to assess the factors related to patient survival. Figure 2 shows the Kaplan‒Meier curves of prognostic factors (weaning from TPN, colon usage, and cholestasis). There were no significant differences according to the presence or absence of weaning from TPN or colon use. A history of cholestasis was identified as a significant prognostic factor (*p* = 0.048) (Fig. 2).

## Discussion

The findings of the present study, based on our experience of treating 14 patients with severe IMD over four decades at a single institution, can be summarized as follows: the underlying disorders causing severe IMD were EAG, IHG, and CIIP, and 50.0% of the patients died; all patients underwent enterostomy involving different lengths and locations depending on the disease; surgery using the distal intestine beyond the stoma was performed in three patients with IHG and one patient with CIIP; cholestasis was the only significant prognostic factor that we identified; 12 of the 14 patients (85.7%) could not be weaned from TPN, and the cause of death was related mainly to TPN and IFALD.

We reported previously that weaning from TPN, without persistent cholestasis, bowel continuity, and residual bowel length > 30 cm, is a predictive factor for a better prognosis and survival for children with SBS [4]. The mortality of pediatric patients with severe IMD related to EAG, IHG, and CIIP in the literature reported from Japan [5–7, 9], is higher than that of pediatric patients with SBS. However, because of the low incidence of the three underlying diseases of severe IMD in this study [6, 9], the clinical data that exist are insufficient for clarifying the prognostic factors. Hence, the accumulation of objective information on cases experienced from each facility is essential for building new evidence.

In our institution’s 39-year experience, pediatric patients with severe IMD were rarely encountered and had a high mortality rate. Most of our patients underwent enterostomy in the neonatal period, excluding those with CIIP who were diagnosed in adolescence. A few patients underwent treatment with the Bishop–Koop method or pull-through to use the colon; however, this was not associated with the prognosis or survival rate. Most of our nonsurvivors had undergone short enterostomy, but this was not in line with the findings of a previous survey, which suggested that jejunostomy at < 50 cm from the ligament of Treitz during the neonatal period is associated with survival, especially for patients with IHG [14]. However, it was difficult to control their severe disorders, and the prognosis was related to other factors as well.

The most common cause of death in our study population was hepatic failure (specifically the condition known as IFALD) caused by long-term TPN. It is estimated that 40–60% of children who receive prolonged TPN are at risk of the development of IFALD, and infants and neonates are at high risk of severe and progressive IFALD [15, 16]. The most important step in the management of IFALD is to promote enteral feeding to reduce the dependence upon PN [17]. Enteral feeding exposes the gastrointestinal tract to nutrient and hormonal stimuli, which are not present when the bowel is kept empty [18]. In our study, the ratio of parenteral to enteral nutrition was not clearly investigated in each patient, whereas cholestasis, which is part of the spectrum of IFALD [16, 19], was the only prognostic factor for severe IMD identified. Other studies have also reported cholestasis to be a significant predictor of survival [6, 11]. Regarding our present and previous study, which analyzed predictors of SBS, cholestasis is a common predictor for patients with SBS and those with severe IMD [4]. This suggests that treating cholestasis is important for all IF patients. The major method used to treat or prevent cholestasis is the administration of fish oil lipid emulsions, such as Omegaven® and SMOFlipid®, which may be most beneficial for infants who cannot tolerate enteral nutrition [20, 21]. A recent study suggests that the neurodevelopment of patients with IF was improved by fish oil emulsion [22]. In our study, only one patient had been given Omegaven®; therefore, the relationship between the administration of fish oil emulsion and mortality remains unclear. However, it is expected that fish oil lipid emulsion will be used extensively in Japan to improve the mortality rate of patients with severe IMD.

The other cause of death was CRBSI, also a complication of long-term TPN [23]. In our previous study on patients with SBS, weaning from TPN was a predictor of survival [4], and found to be important for the prevention of complications. However, weaning from TPN remains challenging for patients with a functional pathogenesis of IF, and in the recent era, long-term TPN failed solely because of catheter complications in 14% of patients [24]. In our study, only two patients (14.3%) could be weaned from TPN. The prevention of CRBSI could reduce morbidity and improve quality of life (QOL), and the loss of venous access sites is an indication for intestinal transplantation (ITx). Therefore, effective therapy for CRBSI is necessary to reduce the incidence of CRBSI and prevent catheter removal. In our institution, therapeutic ethanol lock therapy (ELT) was initiated in 2009 and prophylactic ELT to prevent recurrent CRBSI was introduced from 2012 for patients on home PN. We reported that preventive prophylactic ELT is a safe and effective modality for reducing the need for replacement of central venous catheters for CRBSIs [24]. A recent meta-analysis of ELT for pediatric patients with IF showed that ELT is also improving the recurrence of CRBSI [25]. In this study, the association between ELT and mortality related to severe IMD was not investigated; however, ELT should be considered as a method for improving the mortality rate of patients with severe IMD.

In our country, 40% of cases of ITx are reported to be attributable to IMD [26]. In fact, ITx plays an important role in the treatment of severe IMD; however, only one of our patients underwent ITx, and they died of acute rejection. Global data analyses have reported improving graft and patient survival rates over the last two decades [27, 28]. Results in Japan are comparable with results worldwide and are considered acceptable for the treatment of IF through the development of better immunosuppressive management and understanding [26]. ITx remains the ultimate treatment for patients with irreversible IF who develop life-threatening complications associated with PN. However, it is expected that the number of patients who undergo ITx for severe IMD will increase.

Severe IMD not only presents greater challenges in treatment and management than SBS, but the QOL of these patients is impacted seriously through symptoms such as abdominal pain, bloating, and restrictions on oral intake, and long-term TPN. Our study did not evaluate the relationship between the symptoms and QOL improvement; however, only two patients were able to be weaned off TPN, and the QOL of almost all the patients was not improved. In Japan, QOL after ITx is good [26], so improving the outcomes of ITx for severe IMD will play an important role in improving the QOL of these patients.

Glucagon-like peptide 2 (GLP-2) analog drugs have been approved and launched as a treatment for SBS in Japan, and their usefulness has attracted attention. One study described the efficacy and safety of GLP-2 analogs in infants younger than 1 year of age and provided additional data on children [29]. According to this report, clinically meaningful reductions in parenteral support (PS) volume were observed in infants and children after short- and long-term treatment with GLP-2. Similarly, GLP-2 analogs would reduce the need for PS in patients with severe IMD and the indications for GLP-2 analogs for severe IMD may be expanded through the accumulation of administration experience.

Several limitations associated with the present study warrant mention. First, as the number of patients was small, with low statistical power, our findings will need to be revalidated in a larger cohort. Second, this was a single-center study, and since there is not an established therapeutic strategy for severe IMD in Japan, guidelines have been introduced [23].

## Conclusions

Patients with severe IMDs such as EAG, IHG, and CIIP, still have a high mortality rate, and the prevention or improvement of IFALD is essential for reducing mortality. Cholestasis is a prognostic factor for patients with severe IMD.

## Ethical approval

We have no conflicts of interest to declare in association with the present study.
